# Childhood osteomyelitis-incidence and differentiation from other acute onset musculoskeletal features in a population-based study

**DOI:** 10.1186/1471-2431-8-45

**Published:** 2008-10-20

**Authors:** Øystein Rolandsen Riise, Eva Kirkhus, Kai Samson Handeland, Berit Flatø, Tor Reiseter, Milada Cvancarova, Britt Nakstad, Karl-Olaf Wathne

**Affiliations:** 1 Department of Paediatrics, Ullevål University Hospital, Oslo, Norway; 2 Department of Rheumatology, Rikshospitalet Medical Centre, Oslo, Norway; 3 Department of Radiology, Rikshospitalet Medical Centre, Oslo, Norway; 4 Department of Radiology, Ullevål University Hospital, Oslo, Norway; 5 Department of Biostatistics, Rikshospitalet Medical Centre, Oslo, Norway; 6 Department of Paediatrics, Akershus University Hospital, Nordbyhagen, Norway; 7 University of Oslo, Akershus Faculty Division, Nordbyhagen, Norway; 8 Ministry of Health and Care Services, Oslo, Norway

## Abstract

**Background:**

Osteomyelitis can be difficult to diagnose and there has previously not been a prospective approach to identify all children in a defined geographic area. The aim of this study was to assess the annual incidence of osteomyelitis in children, describe the patient and disease characteristics in those with acute (< 14 days disease duration) and subacute osteomyelitis (≥ 14 days disease duration), and differentiate osteomyelitis patients from those with other acute onset musculoskeletal features.

**Methods:**

In a population-based Norwegian study physicians were asked to refer all children with suspected osteomyelitis. Children with osteomyelitis received follow-up at six weeks, six months and thereafter as long as clinically needed.

**Results:**

The total annual incidence rate of osteomyelitis was 13 per 100 000 (acute osteomyelitis 8 and subacute osteomyelitis 5 per 100 000). The incidence was higher in patients under the age of 3 than in older children (OR 2.9, 95%: CI 2.3–3.7). The incidence of non-vertebral osteomyelitis was higher than the incidence of vertebral osteomyelitis (10 vs. 3 per 100 000; p = .002). Vertebral osteomyelitis was more frequent in girls than in boys (OR 7.0, 95%: CI 3.3–14.7). ESR ≥ 40 mm/hr had the highest positive predictive laboratory value to identify osteomyelitis patients at 26% and MRI had a positive predictive value of 85%. Long-bone infection was found in 16 (43%) patients. ESR, CRP, white blood cell count, neutrophils and platelet count were higher for patients with acute osteomyelitis than for patients with subacute osteomyelitis. Subacute findings on MRI and doctor's delay were more common in subacute osteomyelitis than in acute osteomyelitis patients. Blood culture was positive in 26% of the acute osteomyelitis patients and was negative in all the subacute osteomyelitis patients.

**Conclusion:**

The annual incidence of osteomyelitis in Norway remains high. ESR values and MRI scan may help to identify osteomyelitis patients and differentiate acute and subacute osteomyelitis.

## Background

Haematogenous osteomyelitis is an inflammation of bone and bone marrow, usually caused by bacterial infections, but occasionally caused by fungi, viruses or parasites.[1] Osteomyelitis may cause growth changes or pathological fractures.[2,3]

Acute haematogenous osteomyelitis is usually defined as a history of relevant signs or symptoms of less than 14 days, and subacute haematogenous osteomyelitis as a history of such signs or symptoms of more than 14 days.[4,5] Chronic osteomyelitis evolves over months or years and is characterized by dead bone (sequestrum) and fistulous tracts.[6]

Patients with bone abscesses may have a normal leukocyte count and erythrocyte sedimentation rate (ESR), which makes diagnosis difficult.[7] Bone destruction is not apparent on plain radiographic films until 7 to 10 days after infection.[8] Bone scans are sensitive in the diagnosis of osteomyelitis (73% to 100%), [9-11] but the difficulty in separating bone-marrow processes from soft-tissue disease limits specificity and accuracy.[12] Sensitivity of MRI in the diagnosis of osteomyelitis in adults and children is reported at 88% to 100%, with specificity of 75% to 100%. [12-17]

Studies from Scotland have shown a decline in incidence from 8.7 per 100.000 in 1970 to 2.9 per 100 000 in 1997. The clinical presentation changed from acute to subacute osteomyelitis, and there was a decline in long bone involvement.[3,18] However, a Lithuanian study has shown a rise in the incidence of osteomyelitis from 11.5 per 100 000 in 1982 to 14.3 in 2003.[19] The incidence of vertebral osteomyelitis in children has only been reported from National Patient Registries at < .5 per 100 000.[20,21]

To our knowledge, the incidence of osteomyelitis in children has previously only been reported in retrospective studies and patients with osteomyelitis have only been compared to patients with other acute onset musculoskeletal features in small scale pediatric studies.[14,22] Nor are we aware of any population-based comparative description of patients with acute and subacute osteomyelitis.

We aimed to determine the annual incidence rate of osteomyelitis in children and compare the patient and laboratory characteristics of osteomyelitis patients with those of patients who had other acute onset musculoskeletal features. In addition, we wanted to compare the age, sex, doctor's delay, clinical and MRI characteristics of children with acute and subacute osteomyelitis.

## Methods

### Background population

We conducted a population-based multi-centre study in three counties in South-Eastern Norway (Akershus, Buskerud and Oslo) between May 1, 2004 and June 30, 2005. The total number of children under the age of 16 was 255 303 on January 1, 2004.[23] In Norway the majority of patients receive care in their county of residence, and the homogeneous health care and social security system based on equality of access facilitates recruitment to epidemiological studies.[24]

### Recruitment

The children were examined at county pediatric departments or at the regional department of rheumatology (i.e. at Akershus University Hospital, Buskerud Hospital, Ullevål University Hospital, or Rikshospitalet Medical Centre). All primary care physicians were sent a letter every three months asking them to refer children with possible or evident osteomyelitis or arthritis to the appropriate hospital on the day the patient was first seen. The recruitment criteria were one or more of the following characteristics (under six weeks' duration and not caused by trauma): 1. joint swelling; 2. limited range of motion of one or more joints, walking with a limp or other functional limitations affecting arms and/or legs; 3. pain in one or more joints or extremities together with CRP > 20 mg/L and/or ESR > 20 mm/hr and/or WBC > 12 × 10^9^/L. [25]

At the end of the study, we searched the hospitals' computerized records for 181 relevant diagnoses [based on the International Classification of Diseases, 10th edition (ICD 10) [26]] to identify any patients who met the recruitment criteria but had not been included.

### Inclusion criteria

Only patients younger than 16 who were permanently resident in the participating counties were included. The diagnosis of osteomyelitis was based on the characteristic signs and symptoms of bone infection and one of the following: 1. Positive culture from bone biopsy and/or histology showing inflammation; 2. MRI findings consistent with osteomyelitis; and 3. Positive bone scan if bone biopsy and/or MRI were not done.

### Exclusion criteria

Patients without laboratory examinations and patients who had chronic recurrent multifocal osteomyelitis, sickle cell anemia, fractures or malignant disease were excluded.

### Classification procedure

Follow-up data from medical charts relevant to the final diagnosis were included up to May 2007 (range 22–36 months). Two researchers recorded the clinical information independently on a standardized form. In cases of disagreement, the classification was established in consultation with specialists in pediatric infectious diseases and pediatric rheumatology. Written informed consent was obtained from the parents of the children included in the study. The Regional Ethics Committee for Medical Research and the Ombudsman for Privacy in Research at the Norwegian Social Science Data Services approved the study.

### Classification criteria

Acute osteomyelitis was defined as a history of relevant symptoms of less than 14 days and subacute osteomyelitis as a history of symptoms of 14 days or more.

Arthritis was defined as inflammation of the synovia.[27] Septic arthritis was defined as bacteria cultured from synovial fluid and/or synovial fluid with WBC count > 50 × 10^9^/L.

### Clinical and laboratory assessments

Laboratory, microbiological and radiological tests were performed at each of the hospitals as part of the routine diagnostic procedure. Hemoglobin, WBC with differential, CRP (quantitative turbidimetric or immunoturbidimetric method) and ESR (conventional Westergren method) were assessed on admission. Clinical follow-up was planned for six weeks and six months after admission.

### Radiological tests

Three-phase bone scan (^99m^Tc methylene diphosphonate) was recommended if there was doubt about the localization of the osteomyelitis. MRI was recommended within 3 days if mono- or oligoarthritis of < 2 weeks' duration occurred in combination with one of the following four: 1. fever > 38.5°C; 2. CRP > 30 mg/L or ESR >30 mm/hr or WBC > 12 × 10^9^/L; 3. excessively painful joint or bone; 4. other suspicious factors for osteomyelitis or septic arthritis. We also recommended that MRI should be performed within 14 days if arthritis persisted for more than one week in one to three joints.

Two experienced radiologists retrospectively evaluated the MRIs, blinded to all clinical information except for the patient's age and that there was clinical concern for osteomyelitis. In cases of bone marrow edema, subacute osteomyelitis was defined as well-circumscribed lesions with homogeneous or peripheral contrast enhancement, periosteal inflammation, fibrosis, fistula or sequester. Acute osteomyelitis was defined as a poor interface between the normal and diseased bone marrow. The radiologists also reported the presence of arthritis in a nearby joint, other soft tissue abnormalities, and other orthopedic conditions. The MRIs were performed in different machines in different hospitals (1.0 T or 1.5 T). The MRI examinations had at least one T1 spin echo sequence and one STIR (Short T1 Inversion Recovery) sequence. In most cases (and in every case with well circumscribed lesions), there was also at least one contrast-enhanced T1 spin echo sequence.

One of the radiologists was then informed of the final diagnosis, and evaluated the follow-up plane radiographs and/or MRIs of the osteomyelitis patients in order to report the presence of any remaining signs or sequelae.

### Statistics

Relations between categorical variables were studied using Chi-square test or Fisher's exact test. The continuous variables in our study were not normally distributed. Non-parametric tests were used: the Mann-Whitney-Wilcoxon test for comparison between two groups and the Kruskal-Wallis test for comparison between multiple groups. The continuous variables were described by reference to the median and interquartile range. The sensitivities and specificities of the laboratory tests used to discriminate between patients with and without osteomyelitis were presented graphically using ROC curves. The area under the ROC curve (ROC AUC) provides a measure of the overall discriminative ability of the test. AUC equals .5 when the ROC curve corresponds to random chance, and 1.0 when there is perfect accuracy.[28] P-values of < .05 were considered significant, however, P-values of < .01 were considered significant when we compared more than two groups (Additional file 1). All analyses were performed using SPSS for MS Windows, version 13.

## Results

**Figure 1:**
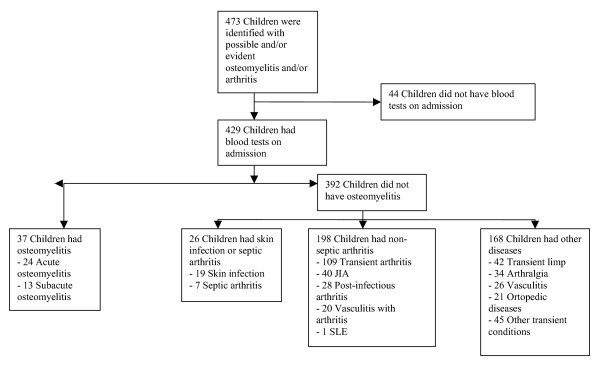
**Flow chart showing recruitment of children with osteomyelitis.** JIA- Juvenile Idiopathic Arthritis; SLE – Systemic Lupus Erythematosus.

Four hundred and twenty-nine (91%) of 473 patients recruited to our study underwent laboratory examinations and were considered eligible for further analysis (Figure 1). Thirty-seven patients had osteomyelitis, 26 had septic arthritis or a skin infection, 198 had non-infectious arthritis and 168 had other conditions. Two hundred and ninety-eight (69%) of the 429 patients were included prospectively, and 131 (31%) were identified by chart review. Thirty-six (97%) of the osteomyelitis patients and 234 (60%) of non-osteomyelitis patients received follow-up at six weeks, and 33 (89%) osteomyelitis patients and 151 (39%) non-osteomyelitis patients received follow-up at six months. The osteomyelitis patients who did not attend the planned follow-up reported (on their previous visit or by telephone) that they did not need further medical care. Non-osteomyelitis patients with persistent symptoms received further specialist health care. There was no difference as regards age, sex and duration of symptoms between the patients who fulfilled the inclusion criteria and had blood tests on admission, and those who were excluded or did not have blood tests (data not shown).

### Incidence of osteomyelitis

The annual incidence rate of osteomyelitis was 13 per 100 000 children under 16 years of age (Additional file 2). The incidence of non-vertebral osteomyelitis was higher than the incidence of vertebral osteomyelitis (10 vs. 3 per 100 000, p = .002). The incidence of osteomyelitis was higher in patients under the age of 3 than in older children (OR 2.9, 95%: CI 2.3–3.7). Vertebral osteomyelitis was more frequent in girls than in boys (OR 7.0, 95%: CI 3.3–14.7).

### Osteomyelitis patients versus children with other acute onset musculoskeletal features

**Figure 2:**
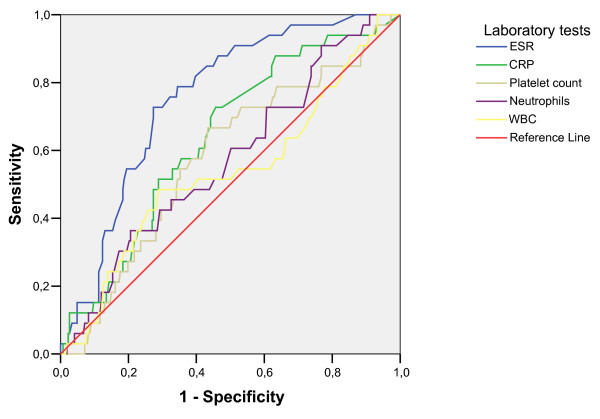
**ROC curves of laboratory tests used during the first visit to discriminate between patients with osteomyelitis (n = 37) and patients without osteomyelitis (n = 392).** The curves plot the relationship between the true positive rate (Sensitivity, y-axis) and the false positive rate (1 - Specificity, x-axis) at different cut-off titers. The higher the cut-off titer that is chosen, the lower the sensitivity and the higher the specificity, and visa versa. The diagonal reference line (area under curve (AUC) = .5), indicates no discrimination. The greater the distance of the curve from the diagonal, the higher the overall discriminative ability of the test.

Figure 2 shows the ROC curves for ESR, CRP, WBC, neutrophils and platelet counts when used to discriminate between patients with and without osteomyelitis. ESR had the highest ROC AUC (.754; 95% CI .680–.828), followed by CRP (.638; 95% CI .545–731).

Among the laboratory variables, ESR ≥ 40 mm/hr had the highest positive predictive value at 26% for identifying patients with osteomyelitis (Additional file 1). ESR < 20 mm/hr had the lowest positive predictive value at 3%.

Nineteen (51%) of the 37 osteomyelitis patients were girls. The median age at presentation was 4.3 years (Additional file 3). In addition to clinical signs and symptoms the diagnosis of osteomyelitis was based on bone biopsy and MRI in 11 patients (2 had a negative culture but were positive on histology), on MRI alone in 24 patients and on bone scan alone in 2 patients (MRI and/or bone biopsy not assessed). One of the patients received corticosteroids due to adrenocortical insufficiency, and another child had C2 immunodeficiency.

Patients with osteomyelitis had a longer history of symptoms than patients with septic arthritis or skin infection or non-septic arthritis (p < .001, p = .001). The period between the first physician visit and the first hospital visit was longer for patients with osteomyelitis than for patients with septic arthritis or skin infection or non-septic arthritis (p < .001, p = .001). ESR and CRP were higher in patients with osteomyelitis than in patients with non-septic arthritis or patients with other conditions (p < .001). The presenting symptom, clinical examination and bone involvement in the osteomyelitis patients are presented in Additional file 4.

### Radiological findings

Plain radiographs of the affected areas were assessed for 35 (95%) of 37 patients with osteomyelitis, and 251 (64%) of 392 non-osteomyelitis patients.

Bone scans showed bone uptake on the location of osteomyelitis in 13 (68%) of the 19 osteomyelitis patients tested, and showed bone uptake in 20 (53%) of the 38 non-osteomyelitis tested. This gave a sensitivity of 68%, specificity of 47%, a positive predictive value of 39% and a negative predictive value of 75%. The six patients with a false negative bone scan (4 total negative, 1 wrong location, 1 only soft tissue uptake) were younger than the patients with a true positive bone scan (age median 1.7, range 1.3–2.5 vs. median 9.2, range 1.1–13.9; p = .018).

Bone marrow edema was found in 57 (45%) of 127 patients using MRI after retrospective evaluation. Bone marrow edema was present in all the 35 osteomyelitis patients who were tested. The MRIs could not exclude osteomyelitis in six (27%) of 22 patients who had bone marrow edema and other diagnosis: three had hand joint arthritis, one had knee joint arthritis, one had post-traumatic pain syndrome, and one had osteoid osteoma. This gave a sensitivity of 100%, specificity of 93%, a positive predictive value of 85% and a negative predictive value of 100%.

#### Acute and subacute osteomyelitis

**Figure 3:**
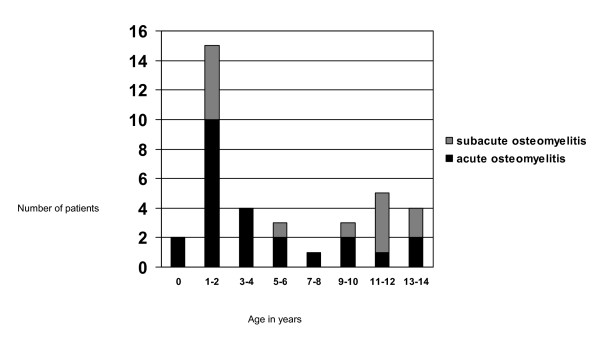
Age on admission of patients with acute osteomyelitis (n = 24) and subacute osteomyelitis (n = 13).

The age distribution of the 24 acute and the 13 subacute osteomyelitis patients is shown in Figure 3. The period between the first physician visit and admission to hospital was longer for patients with subacute osteomyelitis than for patients with acute osteomyelitis (p < .001) (Additional file 5). On admission, the median temperature, ESR, CRP, WBC and neutrophils were higher in patients with acute osteomyelitis than in patients with subacute osteomyelitis (p = .007, p = .012, p = .019, p = .008, p = .003 respectively).

An MRI sign of subacute osteomyelitis was found in 9 (69%) of the patients with subacute osteomyelitis and in 6 (27%) of those with acute osteomyelitis (p = .013).

There was no statistical difference between acute and subacute osteomyelitis patients as regards the presenting symptoms, clinical examination on admission or the anatomic location (data not shown).

#### Treatment and outcome

Thirty-four (92%) of 37 osteomyelitis patients received antibiotics. In 33 (97%) patients, these were administered first intravenously and later orally. The median duration of treatment was 42 days (range 14–137) and the median duration of intravenous treatment was 14 days (range 12–49). Beta-lactamase-resistant penicillin, cloxacillin, was given intravenously to 28 patients (82%) of whom 12 received it in combination with ampicillin. Clindamycin was administered orally to 23 (70%) of 33 patients. For eight patients (24%), the antimicrobials had to be changed due to rash. No patient had Methicillin-resistant *S. aureus*.

Six months after admission, none of the osteomyelitis patients had a history or clinical sign of an ongoing infectious bone process. Thirty-four patients (92%) had radiological follow-up [plain radiograph (n = 23), MRI (n = 17), at a median time of 3 months after admission (range 1 to 37)]. The MRIs revealed reduction or disappearance of bone marrow edema in all patients and sequestrum or fistula in two of the subacute osteomyelitis patients (3 months after admission). The most frequent findings in the nine vertebral osteomyelitis patients were decreased height of vertebral bodies (n = 8), decreased disc space with endplate irregularities on both sides of the disc (n = 7) and pathological angle of the vertebral axis (n = 4). One of the two patients without disc inflammation developed a Schmorl's node. No patients had isolated discitis.

The three osteomyelitis patients who did not receive antimicrobials were identified after reevaluation of the MRIs. These patients recovered clinically within two months, but radiological follow-up showed erosion or sclerosis of the affected bones.

## Discussion

This is the first study that aimed to identify all children with osteomyelitis in a defined geographic area. We found a total annual incidence rate of osteomyelitis in children of 13 per 100 000. Thirty-five percent had subacute osteomyelitis. Osteomyelitis was most common in patients under 3 years of age (28 per 100 000). ESR was the best laboratory test for identifying osteomyelitis patients, but the highest positive predictive value was only 26% (ESR ≥ 40 mm/hr). The most frequent bone involvements were the long bones. Vertebral osteomyelitis was common (24%). Blood cultures were negative for all patients with subacute osteomyelitis and only positive for 26% of patients with acute osteomyelitis. The temperature and the laboratory test results were higher for patients with acute osteomyelitis than for patients with subacute osteomyelitis. Subacute MRI signs were present in 69% of the osteomyelitis patients with disease duration of more than 14 days. The median doctor's delay was 63 days in the subacute osteomyelitis patients.

A limitation of this study is that, due to the recruitment criteria, children with disease duration of more than six weeks and children with trauma may not have been included. We wanted to avoid including children with larger injuries or fractures. However, on admission patients with a history of trauma were not excluded. There are studies indicating that a history of trauma can precede osteomyelitis.[2] As symptoms like back pain and refusal to sit were not among our recruitment criteria, we may not have identified all patients with vertebral osteomyelitis. Although all primary care physicians were repeatedly invited to participate in our study, we have no verification that our request was followed up in every case. In addition, few non-osteomyelitis patients underwent MRI and/or bone scans and/or had follow-up at six weeks and six months. However, the vast majority of patients who did not receive further diagnostic tests or follow-up either received a specific diagnosis or no longer showed symptoms after a few days. The clinical value of testing these patients would probably have been limited. A further limitation was that only 13 (35%) of 37 osteomyelitis patients underwent bone biopsies, which probably led to the low number of patients with a positive microbiological test. Twenty-four (65%) of the osteomyelitis patients were classified on the basis of clinical features and MRI. A poor interface between the normal and diseased bone marrow was found in acute osteomyelitis cases. This is known in the literature.[29] Sensitivity of MRI in the diagnosis of osteomyelitis in adults and children is reported at 88% to 100%, with a specificity of 75% to 100%. [12-17] Assets like fat suppression and contrast enhancement may not help to distinguish infectious from non-infectious inflammatory conditions on MRI.[15] As trauma, acute infarction in sickle cell anemia, recent radiation therapy, osteoid osteoma and medullary tumors may all simulate the signal alterations seen with osteomyelitis, clinical and plain radiograph correlations are essential if MRI is being used for diagnostic purposes.[14,15,30] All of our osteomyelitis patients diagnosed using MRI had a clinical history and plain radiographs that supported the diagnosis.

Bone scans were false negative in six osteomyelitis patients under 3 years of age. Some reports have found a low sensitivity in the very young.[31,32] However, Aigner *et al *have shown that bone scans are highly sensitive in relation to very young children.[33] It is thought that bone scans are positive within the first week [34], and we may have assessed bone scans too early in the disease course. As not all patients were tested with bone scan our data should be interpreted cautiously.

Our total incidence of osteomyelitis at 13 per 100 000 was similar to retrospective studies in Norway and Lithuania (10 to 14 per 100 000).[19,35] The incidence in Maori children was very high at 74 per 100 000.[36] The low incidence in Scotland at 3 per 100 000 could be due to methodology.[18] We believe that a prospective methodology is an asset in identifying mild cases. Thirty-five percent of our patients had subacute osteomyelitis. In the Scottish studies, 50% of patients had subacute osteomyelitis, i.e. there was an increase in the proportion of children with subacute osteomyelitis and a decline in the total incidence of osteomyelitis in children of more than 50% between 1970 and 1997.[3,18] Long bones were affected in 43% of our patients. This was similar to newer series of patients at 33% to 51% [37-39], but lower than older series and a series from South Africa at 75% to 95%.[2,5,35] The development of a higher proportion of subacute osteomyelitis and a lower proportion of long bone involvement could be linked to improved standards of living and hygiene.

Twenty-four percent of our osteomyelitis patients had vertebral involvement (annual incidence of 3 per 100 000). In a retrospective series from the US, vertebral involvement was found in 19% of patients with subacute and chronic osteomyelitis, [37] although it was infrequent in most previous series.[2,20,21] Seven of our patients could have been defined as spondylodiscitis. As none of our patients had an isolated inflammation of the disc we considered the term "discitis" inappropriate in this context. Such cases have been variously diagnosed as osteomyelitis, spondylitis or discitis, and this makes a comparison difficult.[35] There are no verified classification criteria on how to distinguish discitis from vertebral osteomyelitis.[40]

Our osteomyelitis patients had a median age of 4.3 years, which is similar to that found in Scotland, [18] although the patients in Latvia were older, at 10 years.[19] We found an equal distribution of osteomyelitis between the sexes, which was consistent with another Norwegian study.[35] However, in other countries osteomyelitis has been reported to be more frequent in boys.[2,19]

We found that ESR was the best laboratory test for identifying osteomyelitis patients. It was elevated in 83% of patients on hospital admission, and had a median value of 41 mm/hr. In other studies, ESR was elevated in 88% to 92% of osteomyelitis patients on admission, and the rate of positive test and median or mean ESR value depended on whether there patients had the acute, subacute or chronic form.[4,37] The fact that an ESR ≥ 40 mm/hr only had a positive predictive value of 26% in our study confirms that ESR is an unspecific marker for osteomyelitis.[41]

Twenty-six percent of our acute osteomyelitis patients had a positive blood culture, which was lower than in other studies, at 36% to 74%. However, these studies partly recruited patients on the basis of a positive blood culture.[4,19,35,38,39] Blood cultures can only be positive if there is bacteremia at the time the blood is drawn and if sufficient blood is examined. [42,43]In our study, the amount of blood examined for bacteria may have been insufficient. In another study, negative blood culture was found in osteomyelitis patients with small bones and/or non-staphylococcal disease.[42] In line with other studies, *S. aureus *was the most common microbe in our study.[2,4,38] None of our subacute osteomyelitis patients had a positive blood culture. The presence of a positive blood culture has not been described in other studies.[5,37,44]

We classified osteomyelitis patients with disease duration of 14 days or more as subacute osteomyelitis, a definition also adopted in other papers.[4,5] In 69% of these patients, the MRI showed signs of a subacute process.[29,45,46] One of our patients had sequester on admission, a sign of chronic osteomyelitis.[29] In a study by Erdman *et al*, 93% of pediatric and adult osteomyelitis patients (disease duration of more than four weeks) had MRI signs of a subacute process.[14] This could indicate that some patients take more than 14 days to develop subacute MRI signs, or that not all patients develop them.

Why is doctor's delay so common in subacute osteomyelitis? In the acute osteomyelitis patients concomitant septic arthritis was rare however, elevated body temperature and acute phase reactants were more common. Hence these patients seemed to appear more ill. The subacute osteomyelitis patients tended to be older and it is possible that parents and doctors are less concerned about the signs and symptoms these children present. It would have been interesting to know whether the acute phase reactants had been more elevated prior to hospital admission. In Norway, CRP is frequently used as a marker of inflammation in primary care. Perhaps more use of ESR could help to identify patients at an earlier stage.

## Conclusion

The incidence of osteomyelitis in Norway remains high. It was particularly common in children under 3 years of age. There appears to be a decrease in the proportion of patients with acute osteomyelitis and of patients with long bone involvement. Subacute osteomyelitis patients have more moderate laboratory results and a different presentation on MRI than acute osteomyelitis patients. A blood culture is insufficient to identify microbes in most patients.

## Abbreviations

WBC: white blood cell count; CRP: C-reactive protein; ESR: erythrocyte sedimentation rate; MRI: magnetic resonance imaging; ROC: receiver operating characteristics; AUC: area under the curve; JIA: Juvenile idiopathic arthritis; SLE: systemic lupus erythematosus; OR: odds ratio; CI: confidence interval.

## Competing interests

The authors declare that they have no competing interests.

## Authors' contributions

ØR, KH, BF and KOW have contributed substantially to conception, design, analysis, interpretation of data, drafting and revising of the manuscript. EK and TR have evaluated all the MRIs and have substantially contributed to analysis, interpretation of data, drafting and revising. BN and MC have contributed substantially to analysis and interpretation of data and revising of the manuscript. All authors read and approved the final manuscript.

## Pre-publication history

The pre-publication history for this paper can be accessed here:



## Supplementary Material

Additional file 1 **Table 1** Click here for file

Additional file 2 **Table 2** Click here for file

Additional file 3 **Table 3** Click here for file

Additional file 4 **Table 4** Click here for file

Additional file 5 **Table 5** Click here for file
