# Supplementary material for: Childhood osteomyelitis-incidence and differentiation from other acute onset musculoskeletal features in a population-based study
Source: BMC Pediatr. 2008 Oct 20;8:45. doi: 10.1186/1471-2431-8-45 (PMC2588573; doi:10.1186/1471-2431-8-45)
Supplement: Additional file 1 — Table 1 [file 1471-2431-8-45-S1.doc]

**TABLE 1. Annual incidence of various types of osteomyelitis^*^**

**____________________________________________________________________________________**

Total Girls Boys

_________________ __________________ _____________________

n Incidence per n Incidence per n Incidence per

100 000 100 000 100 000

____________________________________________________________________________________

Total osteomyelitis 34 13.3 19 15.2 15 11.5

Location

- non-vertebral 26 10.2^1^ 12 9.6 14 10.7

- vertebral 8 3.1 7 5.6^2^ 1 0.8

Age group

- 0–2 years 14 28.1^3^ 9 37.0 5 19.7

- 3–15 years 20 9.7 10 10.0 10 9.5

Onset type

- acute osteomyelitis 21 8.2 12 9.6 9 6.9

- subacute osteomyelitis 13 5.1 7 5.6 6 4.6

**_____________________________________________________________________________________**

^1^ P = .002 vs. vertebral ^2^ P = .035 vs. boys ^3^ P = .001 vs. age 3–15

^*^ Admitted between June 1, 2004 and May 31, 2005
