# Supplementary material for: Childhood osteomyelitis-incidence and differentiation from other acute onset musculoskeletal features in a population-based study
Source: BMC Pediatr. 2008 Oct 20;8:45. doi: 10.1186/1471-2431-8-45 (PMC2588573; doi:10.1186/1471-2431-8-45)
Supplement: Additional file 2 — Table 2 [file 1471-2431-8-45-S2.doc]

**TABLE 2. Acute phase reactants on admission as diagnostic tests for osteomyelitis in a cohort of**

**children with acute onset musculoskeletal features**

**_____________________________________________________________________________________________________________________________________**

Osteomyelitis Non-osteomyelitis Sensitivity Specificity Positive Negative

ESR (n=35) ESR (n=332) % % predictive predictive

CRP (n=36) CRP (n=388) value % value %

______________________________________________________________________________________________________________________

ESR < 20 6 (17) 199 (60) 17 40 3 82

ESR ≥ 20 29 (83) 133 (40) 83 60 18 97

ESR ≥ 40 19 (54) 53 (16) 54 84 26 95

ESR ≥ 60 5 (14) 23 (7) 14 93 18 91

CRP < 10 14 (39) 257 (66) 39 17 5 75

CRP ≥ 10 22 (61) 131 (34) 61 66 14 95

CRP ≥ 20 18 (50) 99 (26) 50 74 15 94

CRP ≥ 40 9 (25) 55 (14) 25 86 14 93

**_________________________________________________________________________________________________________________________**

Values in brackets are %

ESR = erythrocyte sedimentation rate (mm/hr); CRP = C-reactive protein (mg/L)
