# Supplementary material for: Childhood osteomyelitis-incidence and differentiation from other acute onset musculoskeletal features in a population-based study
Source: BMC Pediatr. 2008 Oct 20;8:45. doi: 10.1186/1471-2431-8-45 (PMC2588573; doi:10.1186/1471-2431-8-45)
Supplement: Additional file 3 — Table 3 [file 1471-2431-8-45-S3.doc]

**TABLE 3. Characteristics of patients with osteomyelitis versus patients with other acute onset musculoskeletal features on admission**

**_________________________________________________________________________________________________________________________________**

Osteomyelitis Septic arthritis P-value vs. Non infectious P-value vs. Other P-value vs.

(n=37) (n=7) or infection septic arthritis or arthritis non infectious (n=168) other

of skin (n = 19) infection of skin (n=198) arthritis

__________________________________________________________________________________________________________________________________

Girls, no. (%) 19 (51) 8 (31) NS 81 (41) NS 82 (49) NS

Age on admission, yrs 4.3 (1.6-14.9) 2.3 (1.1–4.8) NS 5.2 (3.1-7.9) NS 5.5 (3.1-10.1) NS

Duration of symptoms,

days 8 (4–49) 2 (1–6) < .001 3 (1-14) .001 5 (1-47) NS

Duration from first visit

by primary care physician,

days 3 (0–12) 0 (0–0) < .001 0 (0-2) .001 1 (0-3) NS

ESR 41 (27–52) 43 (13–56) NS 15 (7-29) < .001 12 (6-26) < .001

CRP 21 (5–44) 30 (6–67) NS 5 (2-18) < .001 5 (1-16) < .001

**_____________________________________________________________________________________________________________________________________**

All results presented as median (interquartile range) unless otherwise specified

P-values < .01 were considered statistically significant

ESR = erythrocyte sedimentation rate (mm/hr); CRP = C-reactive protein (mg/L); NS = not significant
