# Supplementary material for: Childhood osteomyelitis-incidence and differentiation from other acute onset musculoskeletal features in a population-based study
Source: BMC Pediatr. 2008 Oct 20;8:45. doi: 10.1186/1471-2431-8-45 (PMC2588573; doi:10.1186/1471-2431-8-45)
Supplement: Additional file 4 — Table 4 [file 1471-2431-8-45-S4.doc]

**TABLE 4. Presenting symptom, clinical examination and bone involvement in 37 patients with osteomyelitis**

**___________________________________________________________________________________________________________**

**Presenting symptom**

Refusal to walk and/or sit and/or a limp 22 (59)

Decreased range of motion and/or localized swelling 9 (24)

Localized joint and/or bone pain 5 (14)

**Clinical examination**

Localized tenderness 22 (59)

Localized swelling 15 (41)

Localized temperature increase 6 (16)

Localized erythema 5 (14)

**Involvement of bones**

Single bone involvement 27 (73)

Long bones^1^ 16 (43)

- epiphysis/epiphyseal plate 8 (21) / 6 (16)

- metaphysis/diaphysis 13 (35) / 11 (30)

Craniofacial 1 (3)

Upper extremities 2 (5)

Columna 9 (24)

Pelvis 3 (8)

Lower extremities 22 (59)

- Femur, tibia or fibula 12 (32)

- Foot 10 (27)

**______________________________________________________________________________________________________________________**

Values are no. of patients (%)

^1^ Tibia (n = 6), femur (n = 4), fibula (n = 2), phalange (n = 2), humerus (n = 2), claviculae (n = 1)
