# Supplementary material for: Childhood osteomyelitis-incidence and differentiation from other acute onset musculoskeletal features in a population-based study
Source: BMC Pediatr. 2008 Oct 20;8:45. doi: 10.1186/1471-2431-8-45 (PMC2588573; doi:10.1186/1471-2431-8-45)
Supplement: Additional file 5 — Table 5 [file 1471-2431-8-45-S5.doc]

**TABLE 5. Characteristics in 24 acute and 13 subacute osteomyelitis patients on admission**

**____________________________________________________________________________________________________________________________________________**

Acute Subacute P-value

osteomyelitis osteomyelitis

____________________________________________________________________________________________________________________________________________

**Patient characteristics and laboratory**

**tests**

Girls 12 (50) 7 (54) NS

Age, yrs 2.7 (1.4–8.2) 10.0 (1.7–12.1) NS

Duration from first visit by physician

until first visit to hospital, days 2 (0–5) 63(20–93) < .001

Temperature (°C) 37.7 (37.0–38.3) 37.0 (37.0–37.2) .007

ESR (mm/hr) 44 (36–53) 22 (12–46) .012

CRP (mg/L) 27 (7–50) 6 (4–21) .019

WBC (x 10^9^ cells) 12 (8–13) 8 (7–9) .008

Neutrophils (x 10^9^ cells) 7 (5–8) 4 (3–5) .003

Platelet count (x 10^9^/L) 366 (287–444) 362 (289–423) NS

Concomitant septic arthritis 3 (13) 0 (0) NS

**MRI findings**

Increased signal and/or thick synovia 6/22 (22) 5/13 (38) NS

Subacute process on MRI 6^1^/22 (27) 9^2^/13 (69).013

**Microbiology**

Positive bacterial culture 11^3^/23 (48) 4^4^/12 (25) NS

- Blood 6^5^/23 (26) 0/11 (0) NS

- Bone 4^6^/6 (67) 4^7^/7 (57) NS

- Synovial fluid or soft tissue abscess 3^8^/4 (75) 0/1 (0) NS

_________________________________________________________________________________________________________________________________________

Values are the median, (interquartile range) or the number of patients (%)

^1^ Sharp interface between normal and diseased bone marrow (n = 6), rim of low signal intensity (n = 5), bone cyst (n = 2), and periosteal inflammation with abscess (n = 1). Four of the patients had MRI conducted more than 14 days after the onset of symptoms.

^2^ Sharp interface between normal and diseased bone marrow (n = 9), rim of low signal intensity (n = 6), bone cyst (n = 5), periosteal inflammation (n = 2), sclerosis or fibrosis (n = 2), and sequester (n = 1).^3^ *S. aureus* (n = 6), *S. pneumonia* (n = 2), *S. pyogenes* (n = 2), *Kingella kingae* (n = 1) ^4^ *S. aureus* (n = 3), *S. oralis* (n = 1) ^5^ *S. aureus* (n = 4), *S. pneumonia* (n=1), *S. pyogenes* (n=1) ^6^ *S. aureus* (n=2), *Kingella kingae* (n=1), *S. pyogenes* (n=1) ^7^ *S. aureus* (n=3), *S. oralis* (n=1) ^8^ *S. pneumonia* (n=1), *S. pyogenes* (n=1), *S. aureus* (n=1)

Two of the nine vertebral osteomyelitis patients had positive cultures for *S. aureus*

Acute osteomyelitis: a history of less than 14 days at the time of admission.

Subacute osteomyelitis: a history of 14 days or more at the time of admission.

ESR = erythrocyte sedimentation rate; CRP = C-reactive protein; WBC = white blood cell count; MRI = magnetic resonance imaging
